# Indoor Pollen Concentrations of Mountain Cedar (*Juniperus ashei*) during Rainy Episodes in Austin, Texas

**DOI:** 10.3390/ijerph19031541

**Published:** 2022-01-29

**Authors:** Susanne Jochner-Oette, Johanna Jetschni, Petra Liedl, Annette Menzel

**Affiliations:** 1 Physical Geography/Landscape Ecology and Sustainable Ecosystem Development, Catholic University of Eichstätt-Ingolstadt, 85072 Eichstätt, Germany; johanna.jetschni@ku.de; 2 Munich Institute for Integrated Materials, Energy and Process Engineering, Technical University of Munich, 85748 Garching, Germany; petra.liedl@tum.de; 3 TUM School of Life Sciences, Ecoclimatology, Technical University of Munich, 85354 Freising, Germany; amenzel@wzw.tum.de; 4 Institute for Advanced Study, Technical University of Munich, 85748 Garching, Germany

**Keywords:** indoor pollen, mountain cedar, personal volumetric air samplers, thermal labs, ventilation

## Abstract

Standard pollen monitoring programs evaluate outdoor pollen concentrations; however, information on indoor pollen is crucial for human wellbeing as people spend most of the day in indoor environments. In this study, we investigated the differences in indoor mountain cedar pollen loads between rooms of different uses and with different ventilation at The University of Texas in Austin and focused on the effect of rainy episodes on indoor/outdoor ratios of pollen concentrations. Pollen were sampled outdoors and indoors, specifically in seven rooms and in two thermal labs with controlled ventilation, during the daytime on 6 days in 2015. We calculated daily pollen concentrations, campaign pollen integrals (CPIn, the sum of all daily pollen concentrations) and ratios between indoor and outdoor concentrations (I/O ratio). Pollen concentrations differed substantially based on features related to room use and ventilation: Whereas the highest CPIn was observed in a room characterized by a frequently opened window and door, the smallest CPIn was related to a storeroom without any windows and no forced ventilation. Our results showed that rainy episodes were linked to a higher mean I/O ratio (0.98; non-rainy episodes: 0.05). This suggests that pollen accumulated indoors and reached higher levels than outdoors. Low ratios seem to signal a low level of risk for allergic people when staying inside. However, under very high outdoor pollen concentrations, small ratios can still be associated with high indoor pollen levels. In turn, high I/O ratios are not necessarily related to a (very) high indoor exposure. Therefore, I/O ratios should be considered along with pollen concentration values for a proper risk assessment. Exposure may be higher in indoor environments during prevailing precipitation events and at the end of the pollen season of a specific species. Standardized indoor environments (e.g., thermal labs) should be included in pollen monitoring programs.

## 1. Introduction

Allergies are regarded as an expanding global epidemic [1]. They pose a major risk to human health and imply drastic economic losses by triggering diseases such as allergic rhinitis or even anaphylaxis [2]. The World Health Organization (WHO) reported that 30–40% of the global population is affected by allergies [1]. Regarding the United States, Austin (Texas) is among the 100 worst places for seasonal allergies (rank 61; [3]).

Mountain cedar (*Juniperus ashei* J. BUCHHOLZ; Ashe juniper) is a wind-pollinated shrub or small tree, which produces approx. 400,000 pollen grains per male cone and up to 500 billion pollen grains per individual [4,5]. These pollen are mainly released into the air from mid or late December to February [6]. Mountain cedar pollen are regarded as important aeroallergens [7], especially in the south central United States [8]. The largest populations of mountain cedar can be found along the limestone slopes of the Edwards Plateau in central Texas [9].

A high production and presence of pollen inevitably leads to high atmospheric pollen concentrations: Hourly concentrations higher than 40,000 pollen grains/m^3^ were reported for Austin and Junction, Texas [10]. During three winters in Austin, hourly concentrations above 10,000 pollen grains/m^3^ were recorded 25 times; in Junction, this level was reached 12 times over two winters. For the juniper seasons of 2009 and 2010, a highest hourly concentration of more than 70,000 pollen grains/m^3^ was observed in Junction [11]. Regarding peak levels of daily mean pollen concentration, a value of 5501 pollen grains/m^3^ was measured in Austin in 2015 [12].

During days with high pollen concentrations, allergy-affected people might reduce their symptoms through medication [13] or the avoidance of pollen exposure [14]. One avoidance strategy is to stay indoors where pollen concentrations are expected to be lower than outdoors [15]. Considering the high quantities of outdoor pollen in the case of mountain cedar, it is relevant to assess the pollen exposure for indoor conditions. A deeper knowledge of indoor pollen conditions is also important since worldwide people spend approx. 90% of their time in different indoor environments [16]. The importance of assessing indoor air quality has been pointed out by many authors [17,18,19]. The causal relationship between exposure to pollen allergens and symptoms of allergic reactions [20] suggests that focusing solely on background pollen concentrations is insufficient in terms of a proper allergy treatment.

When examining pollen concentrations indoors, many studies have found lower levels than in outdoor conditions [15,21,22,23,24,25,26,27,28,29,30]. However, some studies also reported that indoor and outdoor concentrations were not correlated (e.g., [22]). In addition to outdoor weather conditions (e.g., precipitation), other factors such as ventilation [31] or the transfer of pollen indoors by people and animals [21] might be important for pollen concentrations in indoor environments.

Therefore, the aim of this study was to investigate indoor pollen concentrations of mountain cedar under high and low outdoor pollen levels and different weather conditions at ten sites in and around a university building in Austin, Texas. We hypothesized that the ratio between indoor and outdoor pollen concentrations differs significantly in relation to the prevailing weather conditions and may show higher values, i.e., indoor pollen levels may reach outdoor levels, during rainy episodes and at the end of the pollen season.

## 2. Materials and Methods

### 2.1. Monitoring Sites and Pollen Measurements

**Figure 1:**
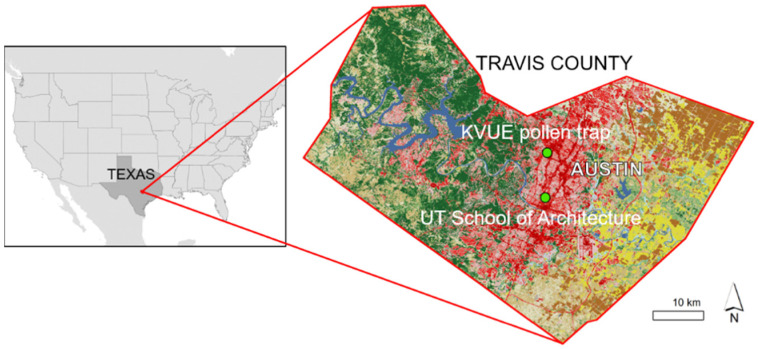
Location of the study site in Texas, USA. (Data: North America political boundaries [32]; Land cover [33]).

**Figure 2:**
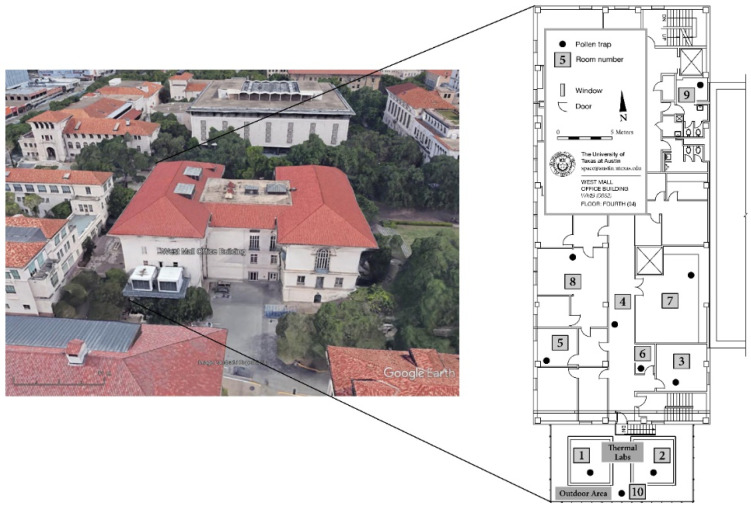
Aerial picture of the West Mall Office Building on the University of Texas at Austin campus with thermal labs on the south of the building (© 2020 Google, CAPCOG); floor plan (fourth floor) with pollen measurement sites (modified after [34]).

Pollen measurements were conducted in Austin, Texas, USA (Figure 1), at ten sites, both inside and outside of the West Hall Office Building (UT School of Architecture), a building on the University of Texas at Austin campus (30.29° N, 97.74° W, 182 m): in six rooms, the hallway, two thermal labs and one outside location (Figure 2). The indoor locations were selected according to obvious differences related to room use and ventilation (see Table 1). All measurement locations were situated on the same altitudinal level/floor (approximately 15 m a.g.l.). The thermal labs (see Figure 2, white “boxes” in front of the building; site numbers 1 and 2) represent an outdoor testing facility, with the primarily research focus on innovative façade design, and allow both full and partial air conditioning. They exemplify typical single office rooms which were set up with comparable ventilation (air change per hour: 0.1) and used in aerobiological research for the first time in the study presented in this paper. One advantage associated with these labs is the opportunity to control ventilation, which makes it possible to assess the accuracy of the method used for pollen trapping.

**Figure 3:**
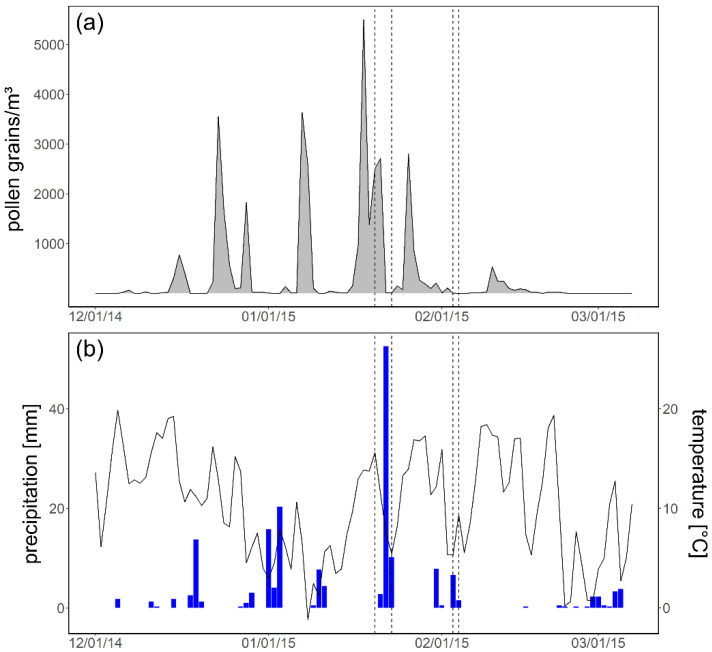
(**a**) *Juniperus* pollen season in 2014/2015 in Austin, with dotted lines indicating the start and end dates of campaign 1 and campaign 2. Pollen data: KVUE and (**b**) corresponding precipitation and temperature data. Data: MesoWest.

Airborne pollen were sampled using personal volumetric air samplers (PVAS, Burkard Manufacturing Co Ltd., Rickmansworth, UK) from 20th to 23rd of January in 2015 (campaign 1) and from 3rd to 4th of February in 2015 (campaign 2) (Figure 3). Air was aspirated at 10 L/min through a horizontally oriented intake at the top of the trap and pollen were deposited on microscope slides coated with Vaseline. These samplers are based on the Hirst principle [35], allowing for a high spatial and temporal resolution and also the adequate representation of indoor conditions. The slides were inserted every second hour during 8 a.m. and 6 p.m. (except on 4 February: 8 a.m.–2 p.m.). In total, we collected, prepared and analyzed 310 samples. Samples were analyzed using light microscopy and all impacted pollen (100% of the impaction area) were counted at ×400 magnification (AXIO Lab.A1, Carl Zeiss Microscopy Deutschland GmbH, Oberkochen, Germany). Counts were converted to concentrations (pollen grains per m^3^ of air). There were no data for six samples (broken microscope slides); in these cases, we calculated the mean value of the previous and following measurements.

Regional pollen information for the whole mountain cedar pollen season was obtained from a monitoring site operated by the KVUE television station in Austin (30.37° N, 97.74° W, 220 m; http://www.kvue.com/weather/allergy-forecast (accessed on 15 May 2015), Figure 1) at a distance of approx. 9 km. Here, pollen were sampled using an Allergenco Air Sampler MK-3 (Environmental Monitoring Systems Inc., Charleston, SC, USA) on a roof at 10 m above ground level. Mean pollen concentrations were provided for a 24-h period in pollen grains/m^3^.

Precipitation and temperature data were obtained from MesoWest at Camp Mabry, Austin (30.32° N, 97.76° W, 204 m).

### 2.2. Vegetation

There are approximately 33.8 million trees in the city of Austin, which cover 30.8% of its area [36]. The most common tree species are mountain cedar (39.9%), cedar elm, live oak, sugarberry and Texas persimmon. However, only 0.7% of mountain cedar can be found in maintained areas such as lawns or parks [36]. No known individuals of mountain cedar were present in the near surroundings of the West Mall Office Building.

### 2.3. Statistical Analyses

We calculated the daily pollen concentration [pollen grains/m^3^] as a mean of all hourly measurements (Equation (1)) and the campaign pollen integral (CPIn), i.e., the sum of the six daily mean pollen concentrations of campaign 1 and campaign 2 (Equation (2)).

(1) $$daily\;pollen\;concentration=\frac{1}{n}\overset{n}{\underset{i=1}{\sum }}hourly\;pollen\;concentration_{i}$$

(2) $$CPln=\overset{6}{\underset{i=1}{\sum }}daily\;mean\;pollen\;concentration_{i}$$

The ratio between indoor and outdoor pollen concentration is denoted as the indoor/outdoor (I/O) ratio and was calculated based on hourly and mean daily pollen concentrations (Equation (3)).

(3) $$I/O\;ratio=\frac{indoor\;pollen\;concentration}{outdoor\;pollen\;concentration}$$

Values greater than 1 indicate that we detected more pollen indoors than outdoors. Campaign 1 was classified as follows to compare non-rainy and rainy episodes: Non-rainy episodes included all samples from 20 January and samples from 21 January until 2 p.m. Rainy episodes included samples from 21 January at 4 p.m. and all the remaining days of campaign 1.

For regional pollen concentrations, we additionally reported the seasonal pollen integral (SPIn), i.e., the sum of all mean daily pollen concentrations within the mountain cedar pollen season [37].

(4) $$SPln=\overset{n}{\underset{i=1}{\sum }}daily\;mean\;pollen\;concentration_{i}$$

Following the recommendations of the European Aeroallergen Network (EAN, www.ean.polleninfo.eu) (accessed on 24 October 2016), the start and the end of the pollen season was defined as the date on which the cumulative sum of daily mean pollen concentration reaches 1% and 95% of the total sum, respectively.

Differences in CPIn or I/O ratios were analyzed using common statistical procedures for comparing means: Since data did not meet the assumption of normal distribution, we used the non-parametric Mann–Whitney U test.

Statistical analyses and visualization were conducted using RStudio (1.3.959) (R Foundation for Statistical Computing, Vienna, Austria) and package ggplot2 [38]. For spatial visualization, we used ESRI ArcMap 10.6.

## 3. Results

The sampling campaigns were conducted during the last third of the *Juniperus* pollen season 2015 (SPIn: 35,857), shortly after the peak value was recorded (5501 pollen grains/m^3^ on 18 January) (Figure 3). Generally, campaign 1 was associated with a much higher regional pollen concentration (20–23 January integral: 5240) than campaign 2 (3–4 February integral: 7).

Weather conditions varied greatly; there was only one day without precipitation (20 January), and the rest of the days were characterized by rainy episodes with maximum values of 52.6 mm (22 January) during campaign 1 and 6.6 mm (3 February) during campaign 2. The mean temperature decreased from 15.1 °C on 20 January to 5.4 °C on 23 January. During the second sampling campaign, mean temperatures were lower, ranging between 5.5 °C (3 February) and 7.2 °C (4 February).

In general, the mean daily pollen concentrations of campaign 2 were much lower than of campaign 1, attributable to the weather conditions and the general progress of the pollen season (Figure 3).

The campaign pollen integral (CPIn), the sum of all daily pollen concentrations within the six sampling days of campaign 1 and campaign 2, was 2787 for the outdoor pollen trap (Table 2), whereas indoor values were 94 to 99% lower. We detected differences in daily *Juniperus* pollen concentrations and in CPIn between rooms with different features, e.g., rooms with or without windows or forced air ventilation. Whereas the window and door of the room with the highest indoor CPIn (161, site 3) was open almost without exception (during non-rainy sampling hours), the smallest CPIn (14) was recorded at site 6, a small storeroom with no window and no forced air ventilation. Air only intruded through the opening of the door for the purpose of operating the pollen trap. Site 9, the female restroom equipped with no window but with ventilation, was also associated with a comparably low CPIn (61). Even smaller values were recorded at sites 1 and 2, the thermal labs (CPIn = 51 and CPIn = 53). Comparison of means (Mann–Whitney U test) revealed no significant differences in hourly pollen concentrations (*p* = 0.723) between these research facilities. The hallway (site 4), linked to a high rate of airflow and change, was associated with the second highest indoor CPIn (138). The rooms that had a high number of people, i.e., potential pollen carriers, entering them (the lecture room, the multiple office) were only associated with medium values (site 7, CPIn = 78; site 8, CPIn = 82).

**Figure 4:**
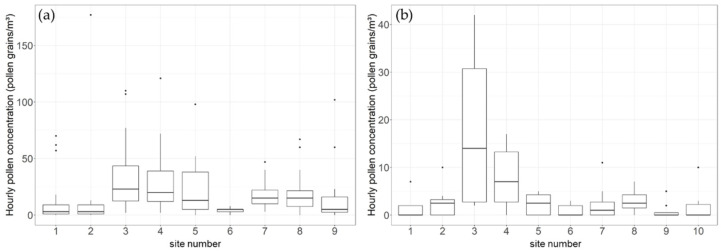
Boxplots based on hourly pollen concentrations [pollen grains/m^3^] for (**a**) campaign 1 (20–23 January) and excl. site 10 (mean/median 749.4/64.0 pollen grains/m^3^) and (**b**) campaign 2 (3–4 February). The interquartile range is represented by the height of the boxes, the maximum and minimum values by the upper and lower whiskers and the median by bold horizontal lines in the boxes; points indicate outliers.

Although mean daily indoor pollen concentrations did not exceed 80 pollen grains/m^3^ (site 3, 20 January), very high hourly pollen concentrations were detected with a maximum of 177 pollen grains/m^3^ at site 2 (12 p.m. on 20 January; Figure 4a). The fluctuation of these hourly values was quite large (campaign 1: highest mean/median 33.0/23.0 pollen grains/m^3^ at site 3, lowest mean/median 3.9/5.0 pollen grains/m^3^ at site 6; campaign 2: highest mean/median 16.6/3.0 pollen grains/m^3^ at site 3, lowest mean/median 1.1/1.0 pollen grains/m^3^ at site 6). Outdoor conditions were associated with a peak value of 3763 pollen grains/m^3^ at 12 p.m. on 20th of January and the mean/median of campaign 1 was 739.4/64.0 pollen grains/m^3^. In campaign 2, these values were much lower (mean/median 1.7/0.0 pollen grains/m^3^).

Table 2 also shows the ratios between daily indoor and outdoor pollen concentration (I/O ratio). Campaign 2 (3–4 February), the period with low background pollen (3 and 1 pollen grains(s)/m^3^, respectively) and precipitation registered, was especially associated with higher pollen concentrations indoors (e.g., 13 pollen grains/m^3^ on 4 January at site 3). Regarding the daily averaged I/O ratio, the highest values were detected for site 3 (I/O ratio = 7.62 (3 February) and I/O ratio = 18.75 (4 February)) and site 4 (I/O ratio = 4.92 (3 February) and I/O ratio = 7.13 (4 February)). In total, 12 out of 58 cases were characterized by a higher indoor *Juniperus* pollen concentration, although daily peak concentrations for indoor conditions related to an I/O ratio >1 were only 31 pollen grains/m^3^ on 22 January (site 9) and 20 pollen grains/m^3^ on 4 February (site 3). Therefore, high I/O ratios alone do not depict the “whole story”. However, it has to be considered that daily means can significantly deviate from hourly values; at site 9, the female restroom, an hourly concentration (6 p.m. on 22 January) of 102 pollen grains/m^3^ was measured. At the same time, the outdoor value was only 7 pollen grains/m^3^, resulting in an hourly I/O of 14.57.

**Figure 5:**
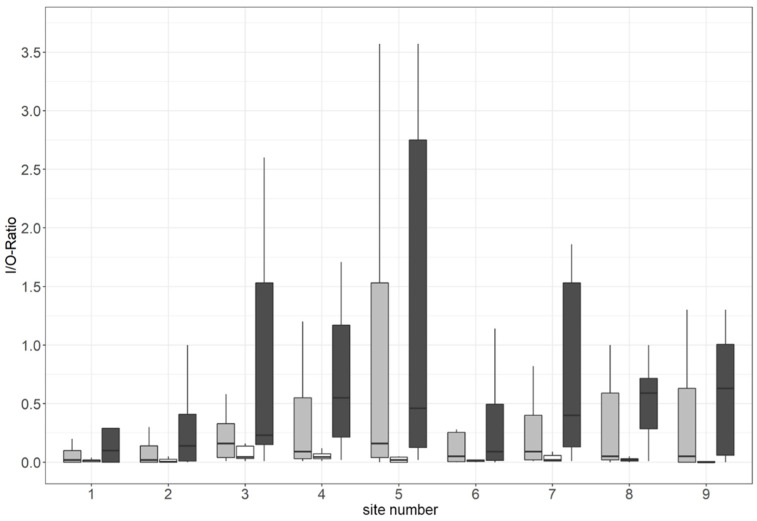
Boxplots showing ratios between hourly indoor and outdoor pollen concentration (I/O ratio) during campaign 1 (20–23 January) for nine sampling sites; grey = all data combined; dark grey = data sampled during rainy episodes (from 21 January 2 p.m. onwards); white = data sampled before the rainy episode; outliers not shown; I/O ratios >1 indicate more pollen indoors than outdoors. The interquartile range is represented by the height of the boxes, the maximum and minimum values by the upper and lower whiskers and the median by bold horizontal lines in the boxes.

When splitting the hourly pollen data of campaign 1 recorded during rainy and non-rainy episodes (Figure 5), higher I/O ratios during rainy episodes (i.e., after 21 January 2 p.m.) were recorded. The mean I/O ratio was 0.98 for rainy episodes and 0.05 for non-rainy episodes. The Mann–Whitney U test confirmed that these differences were statistically significant (*p* < 0.001).

## 4. Discussion

Since mountain cedar pollen are recognized as important aeroallergens in the south central United States [8], it is important to assess the severity of the pollen season. Regarding the conditions in 2015, the severity can clearly be seen in the outdoor concentrations in Austin: e.g., the highest background concentration was 5501 pollen grains/m^3^ on 18 January (see Figure 3) and 2098 pollen grains/m^3^ were obtained by the outdoor PVAS on 20 January (see Table 2). In general, meteorological parameters such as temperature, relative humidity and wind affect pollen release and dispersal and therefore have an influence on pollen concentrations in ambient outdoor air [39]. Besides meteorology, pollen concentrations are also affected by the distance from a pollen source [40] and by vegetation abundance [12]. Although more than one third of Austin’s trees are individuals of mountain cedar [36], their abundance is relatively low (<1%) in maintained areas and not present in the near surroundings of our sampling site. In general, airborne pollen are transported through the air in discrete clouds and homogeneous concentrations are achieved after a considerable time and distance [41]. Therefore, regional scale transport, e.g., via local urban winds, may play an important role and contribute to such high pollen levels. In turn, the presence of trees which individuals are allergic to might not constitute a good indicator for pollen concentrations and hence allergic symptoms, at least when pollen levels of the respective species are that high.

The severity of exposure can also be seen in the hourly outdoor pollen concentrations (outdoor PVAS: 3763 pollen grains/m^3^ at 12 p.m. on 20th of January). On 17 January 2015, prior to the first period of pollen recording in this study (20 to 23 of January), an hourly (10–11 a.m.) peak of 49,533 pollen/m^3^ was recorded at the Lady Bird Johnson Wildflower Center located in the southwest of Austin, where mountain cedar represents the dominant tree species [11]. Levetin et al. [11] documented a peak hourly concentration of 38,022 pollen grains/m^3^ in the pollen season of 1998/1999 in Austin. In addition, Levetin et al. [5] reported 70,367 pollen grains/m^3^ in the season 2009/2010 in Junction. These high hourly peaks suggest that mountain cedar flowering in late winter can induce a high impact on people allergic to cedar pollen in Texas.

To interpret pollen concentrations, the Spanish Aerobiology Network (REA) classifies pollen counts from samples using a threshold system [42]. The group 4 category comprises *Cupressus* pollen. A pollen concentration equal or greater than 51 pollen grains/m^3^ means that a medium percentage of the susceptible population develops symptoms associated with the presence of this type of pollen. Furthermore, it was found that the juniper pollen threshold level causing symptoms in patients living in Turkey is 36 pollen grains/m^3^ [43]. However, thresholds may vary greatly [44], e.g., among patients and geographical regions and in relation to co-occurring pollutants [42] and environmental factors (e.g., meteorology [43]).

For example, it was found that atmospheric conditions with a relative humidity greater than 60% are related to a lower threshold of pollen responsible for triggering symptoms [27]. Although this was reported for grass pollen, relative humidity might also affect the susceptibility of allergic people to other pollen species such as mountain cedar. This could intensify the symptoms indoors and outdoors during rainy episodes even when high pollen concentrations cannot be detected. In our study, a maximum of 80 mountain cedar pollen grains/m^3^ was detected on the first sampling day, the day which was characterized by the highest background pollen concentration. Thus, during high outdoor levels, the absence of symptoms when staying indoors might be doubtable. High hourly values probably exert allergy symptoms, but this might also depend on the time when and the duration people stay inside. High hourly pollen peaks combined with a considerable variability of hourly concentrations suggests that pollen data of a higher temporal resolution are more adequate both for attributing the effects on people being affected by pollen allergies and for interpreting symptom data [12].

In addition, low I/O ratios during dry conditions seem to signal a low level of risk when staying indoors. However, peak values exceeded 5000 pollen grains/m^3^. Thus, small ratios can still be associated with high indoor pollen concentrations. In general, a decline in pollen exposure is expected during rainy weather conditions. This was confirmed for the outdoor and partly also for the indoor pollen sampling sites. Only one room (site 9, the female restroom) was characterized by higher pollen concentrations after the first sampling day (=peak). We demonstrated that there were more pollen inside than outside during rainy episodes, as shown by I/O ratios greater than 1 (see Table 2). Pollen in the outside air decreased, probably due to the washing-off effect and meteorological conditions, which permitted further emissions of mountain cedar pollen. Meanwhile, the fact that pollen were still present indoors led to higher levels being detected there compared to outdoors. High I/O ratios in the second campaign, in which a relatively low background pollen concentration was registered, could provide a hint that pollen were accumulated indoors over the course of the pollen season, reaching even higher levels than outdoors. However, high daily I/O ratios are not necessarily related to a very high indoor exposure. For example, the highest indoor concentration associated to an I/O ratio >1 was 31 pollen grains/m^3^.

Evaluating indoor pollen conditions is of major importance since the knowledge of low(er) indoor pollen levels would enable people to minimize their allergic reactions. Ventilating rooms helps pollen to enter from the outdoor air [31]. This was also confirmed in our study, in which both the single office with an opened window and door and the hallway with a high rate of airflow were linked to the highest CPln. In turn, a lack of ventilation in the storeroom led to the lowest CPln. Pollen grains can also be carried inside by people or pets [21,45] and increase in magnitude when rooms are entered more often and with the increased outdoor activities individuals with access [25,46]. However, the rooms with the highest number of entering people in our study, a lecture room and a multiple office, were only linked to an average CPln. Since the airing of these rooms was mostly limited to short impact ventilation or opening the door for entering, pollen levels probably did not reach higher values. This study was initially planned to present details on the number of entering people but available data, which people were encouraged to enter on a list, were found to be not reliable.

Controlled experiments in terms of the number of entering people should be targeted in future studies. In addition, the need for a standard in assessing indoor pollen becomes obvious when considering the range of pollen concentrations linked to the different measurement sites with different features related to room use and ventilation. The thermal labs, which were primarily developed as testing facilities for innovative façade design (https://soa.utexas.edu/resources/thermal-lab) (access on 26 January 2022), can be adjusted to similar or different rates of air change. Since we found no statistically significant difference between pollen concentrations with the same ventilation settings, we conclude that our method using PVAS is associated with an identical pollen sampling efficiency and that thermal labs can be regarded as prominent testing facilities that should also be used in further aerobiological research. Here, a predefined ventilation and opening towards the average main wind direction at a given location may be prerequisite for generalizing indoor pollen concentrations.

## 5. Conclusions

Evaluating indoor pollen concentrations is essential for human wellbeing since people spend most of their time in different indoor environments. We found that pollen concentrations remained generally low when the airing of rooms was decreased. Low I/O ratios signal a low risk for allergy-affected people while staying inside. However, under high/low outdoor concentrations, small/large ratios can be associated with high/low indoor levels. Therefore, the I/O ratio should be considered, along with pollen concentration values, for a proper risk assessment. An enhanced indoor exposure to mountain cedar pollen during rainy conditions and at the end of the season indicated the accumulation of pollen. Thus, we suggest that outdoor activities should be preferred during these periods. The need for a standard for the assessment of indoor pollen becomes clear when one considers the range of pollen concentrations associated with the different measurement locations with different characteristics in terms of room use and ventilation.

## Figures and Tables

**Table 1:** Overview of the ten different sites with their special features regarding use and ventilation.

Site	Location	Features
1	Thermal Lab 1	Air change per hour: 0.1
2	Thermal Lab 2	Air change per hour: 0.1
3	Office 1	Open door and open window during non-rainy period measurements
4	Hallway	High rate of air flow and change
5	Single Office	No direct connection to hallway, windows frequently opened
6	Storeroom	No window, no forced ventilation
7	Lecture Room	High number of entering people
8	Multiple Office	No direct connection to hallway, windows frequently opened
9	Restroom	No window, but with ventilation
10	Outside	15 m a.g.l.

**Table 2:** Mean daily pollen concentration (p. c.) [pollen grains/m^3^] (rounded figures) for ten sites and six days in 2015 and associated ratios between daily indoor and outdoor pollen concentration (I/O; ratios > 1 indicate more pollen indoors than outdoors) as well as campaign pollen integral (CPIn; sum of six daily values), NA missing data.

Site	Location	Campaign 1								Campaign 2				CPI
		20.01.		21.01		22.01.		23.01.		03.02.		04.02		
		p. c.	I/O	p. c.	I/O	p. c.	I/O	p. c.	I/O	p. c.	I/O	p. c.	I/O	
1	Thermal Lab 1	36	0.02	4	0.02	6	0.18	3	0.01	2	0.69	1	0.75	51
2	Thermal Lab 2	37	0.02	2	0.01	4	0.13	6	0.01	4	**1.46**	1	**1.13**	53
3	Office 1	80	0.04	18	0.10	12	0.38	19	0.04	20	**7.62**	13	**18.75**	161
4	Hallway	61	0.03	21	0.12	19	0.62	19	0.04	13	**4.92**	5	**7.13**	138
5	Single Office	NA	NA	10	0.05	25	0.82	37	0.08	3	**1.15**	1	**1.50**	76+
6	Storeroom	NA	NA	3	0.02	4	0.12	5	0.01	1	0.38	1	**1.88**	14+
7	Lecture Room	31	0.01	12	0.06	16	0.51	13	0.03	6	**2.38**	1	0.75	78
8	Multiple Office	33	0.02	7	0.04	23	0.73	14	0.03	4	**1.46**	1	**1.50**	82
9	Restroom	5	0.00	6	0.03	31	**1.01**	17	0.04	2	0.92	0	0.00	61
10	Outdoors	2098		183		31		472		3		1		2787

Bold values indicate I/O ratios > 1.

## Data Availability

Data sharing not applicable.
